# Distribution of Free and Bound Phenolic Compounds in Buckwheat Milling Fractions

**DOI:** 10.3390/foods8120670

**Published:** 2019-12-12

**Authors:** Beatriz Martín-García, Federica Pasini, Vito Verardo, Ana María Gómez-Caravaca, Emanuele Marconi, Maria Fiorenza Caboni

**Affiliations:** 1 Department of Analytical Chemistry, Faculty of Sciences, University of Granada, Avd. Fuentenueva s/n, 18071 Granada, Spain; bearu15@correo.ugr.es (B.M.-G.); anagomez@ugr.es (A.M.G.-C.); 2 Department of Agricultural and Food Sciences, University of Bologna, Piazza Goidanich 60, (FC) 47521 Cesena, Italy; federica.pasini5@unibo.it (F.P.); maria.caboni@unibo.it (M.F.C.); 3 Department of Nutrition and Food Science, University of Granada, Campus of Cartuja, 18071 Granada, Spain; 4 Institute of Nutrition and Food Technology ‘José Mataix’, Biomedical Research Center, University of Granada, Avda del Conocimiento sn., 18100 Armilla, Granada, Spain; 5 Dipartimento Agricoltura, Ambiente e Alimenti, Università del Molise, via De Sanctis s/n, I-86100 Campobasso, Italy; marconi@unimol.it; 6 Interdepartmental Centre for Agri-Food Industrial Research, Alma Mater Studiorum, Università di Bologna, via Quinto Bucci 336, 47521 Cesena (FC), Italy

**Keywords:** free and bound phenolic compounds, buckwheat flours, HPLC–MS, milling fractions

## Abstract

Buckwheat is a rich source of phenolic compounds that have shown to possess beneficial effect to reduce some diseases due to their antioxidant power. Phenolic compounds are present in the free and in the bound form to the cell wall that are concentrated mainly in the outer layer (hull and bran). Hull is removed before the milling of buckwheat to obtain flours. In order to evaluate the phenolic composition in dehulled buckwheat milling fractions, it was carried out a determination of free and bound phenolic compounds in dehulled whole buckwheat flour, light flour, bran flour, and middling flour by high-performance liquid chromatography-mass spectrometry (HPLC–MS). The most abundant free phenolic compounds were rutin and epiafzelchin–epicatechin-*O*-dimethylgallate, whereas the most abundant bound phenolic compounds were catechin and epicatechin in all buckwheat flours. Besides, the highest content of free phenolic compounds was obtained in bran flour (1249.49 mg/kg d.w.), whereas the greatest bound phenolic content was in middling (704.47 mg/kg d.w.) and bran flours (689.81 mg/kg d.w.). Thus, middling and bran flours are naturally enriched flours in phenolic compounds that could be used to develop functional foods.

## 1. Introduction

Buckwheat (*Fagopyrum esculentum* Moench) as a traditional pseudocereal crop which belongs to the Polygonaceae is extensively utilized as food and as a medicinal plant [1]. Buckwheat is a rich source of starch, protein, and vitamins [2]. In addition, buckwheat is well known for containing phenolic compounds, including phenolic acids such as protocatechuic, syringic acid, and caffeic acid and flavonoids such as rutin (quercetin 3-rutinoside), quercetin, hyperoside (quercetin 3-*O*-b-d-galactoside), quercitrin (quercetin 3-*O*-a-l-rhamnoside), epicatechin, orientin, vitexin, isovitexin, and isoorientin [3,4,5]. Rutin is the most concentrated phenolic compound in Tartary and some common buckwheats, which have a content higher than most other plants [2]. Phenolic compounds in buckwheat have shown to possess antioxidant activity which has been associated with a lower incidence of cardiovascular disease, cancers, and age-related degenerative process [6,7,8,9,10]. 

Phenolic compounds in buckwheat are present in the free and in the bound form to cell wall [11], however, the majority of phenolic compounds are present in the free form, which has a distribution and concentration that is different in each part of the grain: pericarp (hull, husk), coat, endosperm, embryo with axis, and two cotyledons [12]; phenolic compounds are concentrated in the outer layers (hull and bran) of buckwheat grain [2]. Nevertheless, during buckwheat seeds processing into flour, the hull (17–20% of buckwheat grain) is removed by stone dehuller. The resulting product, called groat (intact achene), is milled into bran flour (10–24%), which is a by-product that it is not commonly used in foods, and light flour (55–70%), which consists principally of endosperm and is used in human nutrition [13]. In addition, middling is a by-product from buckwheat milling that is not a flour that comprises different fractions and it includes 12% of the original grain, consisting of fractions of endosperm, bran, and germ [14]. Milling techniques used in the food industry employ mechanical force to break the grains into smaller fragments or fine particles. [15]. Previous studies reported the use of roller milling process in dehulled whole buckwheat to obtain a flour and the separation of this flour into various fractions from outer to inner parts [2,16]. These studies have shown that outer layers are richer in protein, lipid, dietary fiber, and ash content than the inner layers. Also, the antioxidant capacity in flour fractions in the outer layers is higher than that in the inner layers by the increase of phenolic compounds from bran [2,16]. In addition, it has reported that milling fractions that contain outer layers possess a higher concentration of phenolic compounds than whole grain and groat flour fractions [6]. Therefore, the aim of this work was the determination of free and bound phenolic content in different buckwheat meals/flours: whole grain flour, light flour, bran meals, and middling flour in order to evaluate the phenolic concentration in each buckwheat meal fraction. These analyses will furnish new information about the total content of phenolic compounds in each fraction, taking into account the free or extractable fraction and bound or nonextractable phenolic fraction (NEPP). For that purpose, phenolic compounds were extracted and then were analyzed by high-performance liquid chromatography-mass spectrometry (HPLC-MS).

## 2. Materials and Methods 

### 2.1. Sample

**Figure 1:**
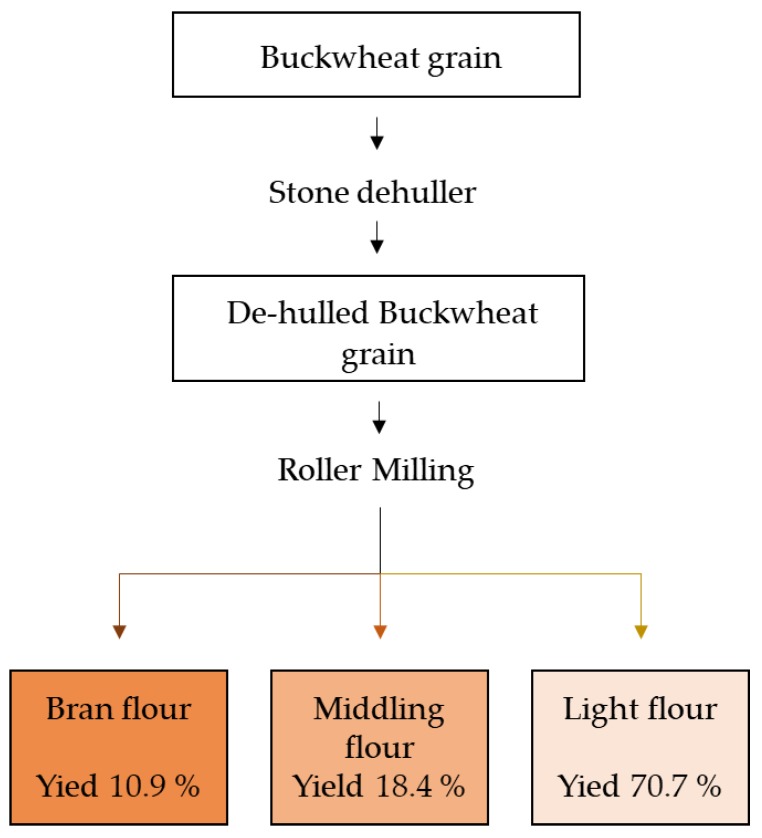
Flowchart of the milling process used for the production of buckwheat flours.

Buckwheat meals/flours were obtained from whole buckwheat grain (cv. Darja) harvested in Matrice (Italy) (41°37′00″ N 14°43′00″ E), situated in a hilly location at 750 m above sea level. The field presented high tenacity of the soil due to the presence of clay. Harvesting took place on September 2018. The grain was dehulled by stone dehuller (GRANO 200 SCHNITZER Stein-Getreidemuhle, Offenburg, Germany), and the groat (dehulled grain) was roller-milled by using an experimental mill (Labormill 4RB Bona, Monza, Italy). This mill is able to produce three milling fractions with different particle sizes that constituted the basis for differentiation between bran meal, middling flour, and light flour (Figure 1). In the bran meal, the majority of particles were >505 μm, while in middling flour, between 219–363 μm, and in light flour, <183 μm. Granulometry analysis was performed using an automatic sieve (Buhler ML1-300, Uzwil, Switzerland).

### 2.2. Reagents and Chemicals

HPLC-grade acetonitrile, water, methanol, acetone, acetic acid, ethanol, hexane, ethyl acetate, diethyl ether, and hydrochloric acid were purchased from Merck KGaA (Darmstadt, Germany). Hydroxide sodium was from Fluka (Buchs, Switzerland). Ferulic acid, catechin, quercetin, and rutin (Sigma-Aldrich (St. Louis, MO, USA)) were used for the calibration curves.

### 2.3. Extraction Method

Extraction of free phenolic compounds from buckwheat flour fractions has been carried out according with the method established by Hung & Morita (2008) [2] with certain modifications. One gram of buckwheat flour was extracted thrice in an ultrasonic bath with a solution of ethanol/water (4:1 *v*/*v*). The supernatants were collected, centrifugated at 2500 rpm for 10 minutes, evaporated, and reconstituted with 1 mL of methanol/water (1:1 *v*/*v*). The extracts were stored at −18 °C until use.

Extraction of bound phenolic compounds was carried out according to the method established by Verardo et al. (2011) [5]: residues of free phenolic extraction were digested with 25 mL of 1M NaOH at room temperature for 18 h by shaking under nitrogen gas. The mixture was acidified (pH 2.2–2.5) with hydrochloric acid in a cooling ice bath and extracted with 250 mL of hexane to remove the lipids. The aqueous solution was extracted five times with 50 mL of 1:1 diethyl ether/ethyl acetate (*v*/*v*). The organic fractions were collected and evaporated at 40 °C in a rotary evaporator. The dry extract was reconstituted in 1 mL of methanol/water (1:1 *v*/*v*) and stored at −18 °C until use.

### 2.4. Determination of Free and Bound Phenolic Compounds by HPLC–MS

A liquid chromatography apparatus HP 1100 Series (Agilent Technologies, Palo Alto, CA, USA) equipped with a degasser, a binary pump delivery system, and an automatic liquid sampler and coupled to a single quadrupole mass spectrometer detector was used. Separation of free and bound phenolic compounds from buckwheat flour fractions was carried out using a C-18 column (Poroshell 120, SB-C18, 3.0 × 100 mm, 2.7 μm from Agilent Technologies, Palo Alto, CA, USA). The gradient elution was the same as that previously established by Gómez-Caravaca et al. [17] using as a mobile phase A acidified water (1% acetic acid) and as mobile phase B acetonitrile. MS analysis was carried out using an electrospray ionization (ESI) interface in negative ionization mode at the following conditions: drying gas flow (N_2_), 9.0 L/min; nebulizer pressure, 50 psi; gas drying temperature, 350 °C; capillary voltage, 4000 V. The fragmentor and m/z range used for HPLC–ESI/MS analyses were 80 V and m/z 50–1000, respectively. Data were processed by the software MassHunter Workstation Qualitative Analysis Version B.07.00 (Agilent Technologies, Santa Clara, CA, USA).

### 2.5. Statistical Analysis 

The results of quantification reported in this work are the averages of three repetitions (*n* = 3). Tukey’s honest significant difference multiple comparison (one-way ANOVA) at the *p* < 0.05 level was evaluated by using the Statistica 7.0 software (StatSoft, Tulsa, OK, USA)

## 3. Results and Discussion

### 3.1. Analytical Parameters of the Method 

An analytical validation of the method was performed considering linearity and sensitivity. In order to quantify phenolic compounds in buckwheat fractions, five calibrations curves were elaborated with the standards ferulic acid, catechin, quercetin, gallic acid, and rutin. Table 1 includes the analytical parameters of the standards used, containing calibration ranges, calibration curves, determination coefficients, limit of detection (LOD), and limit of quantification (LOQ). 

Calibration curves were carried out by using the peak areas analyte standard against the concentration of the analyte for the analysis by HPLC. The external calibration of the standards was elaborated at different concentration levels from LOQ to 100 mg L^−1^. All calibration curves revealed good linearity among different concentrations, and the determination coefficients were higher than 0.9994 in all cases. The method used for analysis showed LOD within the range 0.0040–0.0136 mg L^−1^, the LOQ were within 0.0134–0.0452 mg L^−1^.

### 3.2. Identification of Phenolic Compounds from Buckwheat Extracts by HPLC–MS

Free and bound phenolic compounds in buckwheat flour fractions extracts were analyzed by HPLC with MS detection, and the identification of these compounds was carried out by comparison of molecular weight in bibliography and when available, by co-elution with commercial standards.

A total of 25 free phenolic compounds were identified in buckwheat flours, among them five were phenolic acids and 20 were flavonoids, and they were previously identified in other works [4,18] (Table 2). 

Twenty-four bound phenolic compounds were identified in buckwheat flours: seven were phenolic acid derivatives and 17 were flavonoids, which were identified in previous works (Table 3) [5,18].

### 3.3. Quantification of Free and Bound Phenolic Compounds in Buckwheat Fractions 

Free phenolic compounds were quantified through of calibration curves of standards. A total of 25 free phenolic compounds were quantified in buckwheat meals/flours: de-hulled grain meal, bran meal, middling flour, and light flour (Table 4). 

The most concentrated free phenolic compound in all buckwheat flours was epiafzelchin–epicatechin-*O*-dimethylgallate, whose content was 13.11 mg/kg d.w. in light flour, 93.83 mg/kg d.w. in de-hulled grain meal, 176.67 mg/kg d.w. in middling flour, and 216.94 mg/kg d.w. in bran meal. The second most concentrated in buckwheat flours was rutin, whose content was from 7.03 mg/kg d.w in light flour, 87.33 mg/kg d.w. in de-hulled grain meal, 148.63 mg/kg d.w. in middling flour, to 214.99 mg/kg d.w in bran meal. Thus, the most abundant free flavonoids are present in buckwheat bran meal, followed by middling flour, de-hulled buckwheat meal, and light flour. Besides, 2-hydroxy-3-*O*-β-d-glucopyranosylbenzoic and protocatechuic-4-*O*-glucoside acid appear in buckwheat fractions in significant quantities, whose values were 2.67–2.93 mg/kg d.w. in light flour, 32.71–65.56 mg/kg in de-hulled grain meal, 42.17–79.69 mg/kg d.w. in bran meal, and 78.22–120.56 mg/kg d.w. in middling flour. Therefore, the highest content of phenolic acids appears in middling flour, followed by bran meal, de-hulled grain meal, and light flour. The third most abundant phenolic compound in middling and de-hulled grain meal was protocatechuic-4-*O*-glucoside acid (120.59 and 65.56 mg/kg d.w.), whereas in light flour was swertiamacroside (4.23 mg/kg d.w.), and in bran meal was epicatechin-*O*-3,4-dimethylgallate (98.07 mg/kg d.w.). 

The total free phenolic content in buckwheat flours was decreasing in the following order: bran meal > middling flour > de-hulled buckwheat meal > light flour (1242.49, 901.10, 520.74, and 46.36 mg/kg d.w.). These results are due to the most abundant free phenolic compounds being flavonoids, which corresponded to 66–79% of total free phenolic compounds, and these are found in higher concentration in outer layers than in inner layers of buckwheat grain [2]. For that reason, bran meal contains the highest content of free phenolic compounds, followed by middling flour, as it contains seed coat.

The concentration of free phenolic compounds obtained in buckwheat was compared with that obtained previously in other works. Verardo et al. (2011) [5] quantified the individual free phenolic compounds in de-hulled buckwheat grain, where rutin was the most concentrated, whose value was 35.12% higher than that obtained in the present work and total content of free phenolic compounds was 48.39% higher than in the present work. Nevertheless, the most concentrated free phenolic compound in our work was epiafzelchin–epicatechin-*O*-dimethylgallate, whose value was 50% higher than that obtained by Verardo et al. (2011) [5]. These differences of concentration could be due to the different buckwheat cultivar. Besides, Inglett et al. (2011) [18] quantified the free flavonoid content in different buckwheat flours (fancy, farinetta, supreme, and whole), fancy corresponded with light flour, supreme flour is similar to bran meal, farinetta consists of a fine granulated mixture of aleurone layer of hulled achene and achene embryo, a composition similar to middling flour [19,20]. The value of free flavonoids obtained in our study in light flour, de-hulled grain meal, bran meal, and middling flour (34.47 mg/kg d.w., 371.25 mg/kg d.w., 982.23 mg/kg d.w., and 598.83 mg/kg d.w) were in the same order of magnitude than that obtained in fancy (71.40 mg/kg d.w.), whole buckwheat flour (417.03 mg/kg d.w.), supreme (525.27 mg/kg d.w.), and farinetta (671.50 mg/kg d.w.) by Inglett et al. (2011) [18].

Hung et al. (2008) [2] reported the content of rutin in the free form obtained in different buckwheat flour fractions, and its concentration was 2.5–3 mg/kg d.w. in the innermost layers, whereas in the outer layers, it was 274-337.8 mg/kg. These results were similar to those obtained in the present work in the light flour (7.03 mg/kg dw.) and bran meal (214.99 mg/kg d.w.). Kalinová et al. (2019) [21] reported the free phenolic compounds in the seed coat (553.18 mg/kg d.w.), in the endosperm (2.59 mg/kg d.w.), and in the groat (139.66 mg/kg d.w.). These values were lower than those obtained in bran meal, light flour, and de-hulled grain meal, and also, the content of rutin in seed coat (54.23 mg/kg d.w.) represents a quart of the phenolic bran meal (214.99 mg/kg d.w.) obtained in our study. This could be due to the different cultivar and/or the different methodology of determination of phenolic compounds (by MS detection a higher number of compounds are determined).In addition, Liu et al. (2019) [22] reported the concentration of rutin in common buckwheat (62.19 mg/kg d.w.) that was in the same order as that obtained in de-hulled grain meal (87.33 mg/kg d.w.). According to the results obtained in these previous works, it has shown that rutin in the free form is concentrated in the outer layers, which is in concordance with our results. 

The Table 5 reports the content of bound phenolic compounds in buckwheat flours. Bound phenolic compounds composition in buckwheat flours was similar than that obtained in free phenolic fraction; nevertheless, flavonoids such as isorientin, epiafzelchin–epiafzelchin–epicatechin, Procyanidin B2-dimethylgallate, hyperin, and (epi)afzelchin-(epi)catechin were not detected in bound fraction, whereas some phenolic acids such as syringic and p-coumaric acid, procyanidin A, and myricetin were determined only in bound fraction.

Catechin was the most concentrated bound phenolic compound in all buckwheat flours, representing 25–30% of total bound phenolic compounds, and its concentration was 54.67 mg/kg d.w. in light flour, 95.45 mg/kg d.w. in de-hulled grain meal, 200.17 mg/kg d.w. in middling flour, and 207.74 mg/kg d.w. in bran meal, respectively. The second component most abundant was epicatechin, whose content was 34.67 mg/kg d.w. in light flour, 41.55 mg/kg d.w. in de-hulled grain meal, 59.08 mg/kg d.w. in bran flour, and 97.50 mg/kg d.w. in middling flour. The third most abundant phenolic compound in de-hulled grain meal and bran meal was syringic acid (35.62 mg/kg d.w. and 85.86 mg/kg d.w.), whereas in middling flour it was caffeic acid hexose (56.73 mg/kg d.w.), and in light flour it was swertiamacroside. 

The total bound phenolic content in buckwheat flours was increasing in the following order: light flour < de-hulled grain meal < bran meal < middling flour (207.74, 389.51, 689.81, and 704.47 mg/kg d.w.). Therefore, the highest concentration of bound phenolic compounds is in middling and bran meal due to these compounds being linked to the cell wall of buckwheat layers. Flavonoids represented 59–65% of the bound phenolic fraction. Whereas, phenolic acids represented 35–41% of bound phenolic fraction.

Concentrations of catechin, epicatechin, syringic, and total bound phenolic compounds in de-hulled whole buckwheat flour obtained by Verardo et al. (2011) [5] were 23.88%, 48.54%, and 53.18% higher than those obtained in the present work. Inglett et al. (2011) [18] reported the content of total bound flavonoid in buckwheat flour fractions obtained was 59.25 mg/kg d.w. in fancy, 389.68 mg/kg in farinetta, 530.21 mg/kg in supreme, and 613.77 mg/kg d.w. in whole flour, which are in the same order of magnitude as that obtained in our work. Nevertheless, in this study, the highest bound phenolic content was obtained in whole buckwheat flour, whereas in our work, the maximum value of phenolic content corresponded with the middling flour. This could be due to the different cultivar or because Inglett et al. (2011) [18] could include the hull in the buckwheat grain flour.

The total content of flavonoids was from 157.84 mg/kg d.w. in light flour to 1422.52 mg/kg d.w. in bran meal, whereas the content of phenolic acids was from 96.261 mg/kg d.w. in light flour to 548.63 mg/kg d.w. in middling flour. Total phenolic content was from 254.10 mg/kg d.w. in light flour to 1932.30 mg/kg d.w. in bran meal (Table 6). According to the results, the total phenolic content was increasing in the following order: light flour < de-hulled grain meal < middling flour < bran meal Therefore, middling flour and bran meal possess the highest phenolic content due to bran and the aleurone layer being richer in many phenolic compounds than the others buckwheat flours [23]. Total flavonoid obtained in de-hulled grain meal, bran meal, and middling flour was 49.22%, 71.21%, and 27.83% higher than that obtained in whole grain meal, supreme, and farinetta by liquid chromatography-electrospray ionization- mass spectrometry (LC–ESI-MS) [18]. According to Guo and co-workers, free rutin was determined in a range of 51–81% [24].

## 4. Conclusions

An HPLC–MS has been used for the determination of free and bound phenolic compounds in buckwheat flours: middling flour, bran meal, light flour, and whole meal. The results of this study have shown that the total free phenolic compounds are found in the highest concentration in bran meal, whereas the bound content of phenolic compounds are concentrated in middling flour and bran meal. In buckwheat flours, the main flavonoids were rutin and epiafzelchin–epicatechin-*O*-dimethylgallate, which had the greatest content in bran meal. By contrast, catechin and epicatechin were the main bound flavonoids in buckwheat meal/flours that existed in the greatest quantities in middling and bran fours. 

To conclude, the bran meal and middling flour could be considered as flours enriched in phenolic compounds that could be used to elaborate food with health benefits. Moreover, it has been proved, as the distribution of some phenolic compound varied from bran to middling fraction.

## Figures and Tables

**Table 1:** Analytical parameters of the method proposed.

Standards	Calibration Ranges (mg/L)	Calibration Curves (mg/g)	*r* ^2^	LOD (mg/L)	LOQ (mg/L)
Ferulic acid	LOQ-100	*y* = 119572*x* + 16157	0.9995	0.0136	0.0452
Catechin	LOQ-100	*y* = 170925*x* + 8609.5	0.9994	0.0095	0.0316
Quercetin	LOQ-100	*y* = 402162*x* + 44862	0.9996	0.0040	0.0134
Gallic acid	LOQ-100	*y* = 123892*x* − 4971.6	0.9984	0.0131	0.0437
Rutin	LOQ-100	*y* = 199694*x* − 2067.2	0.9999	0.0081	0.0271

LOD: Limit of detection, LOQ: Limit of quantification.

**Table 2:** Identification table of free phenolic compounds in buckwheat flours.

Peak	Retention Time	[M-H]	Molecular Formula	Compound	In Source Fragments
1	2.1	315	C_13_H_15_O_9_	2-hydroxy-3- *O*-β-d-glucopyranosylbenzoic acid	153
2	2.6	315	C_13_H_15_O_9_	Protocatechuic-4-*O*-glucoside acid	153
3	3.3	451	C_21_H_23_O_11_	Catechin-glucoside	289
4	4.1	341	C_15_H_17_O_9_	Caffeic acid hexose	179
5	4.2	289	C_15_H_13_O_6_	Catechin	
6	4.4	487	C_21_ H_27_ O_13_	Swertiamacroside	179
7	5.0	179	C_9_H_7_O_4_	Caffeic acid	
8	5.5	289	C_15_H_13_O_6_	Epicatechin	
9	6.2	561	C_30_H_25_O_11_	(Epi)afzelchin-(epi) catechin isomer A	543, 289, 271, 435
10	6.8	447	C_21_H_19_O_11_	Orientin	
11	7.0	447	C_21_H_19_O_12_	Isorientin	
12	7.8	431	C_21_H_19_O_10_	Vitexin	
13	7.9	609	C_27_H_29_O_16_	Rutin	
14	7.9	441	C_22_H_17_O_10_	Epicatechin-gallate	289
15	8.0	833	C_45_H_37_O_16_	Epiafzelchin–epiafzelchin–epicatechin	
16	8.2	487	C_21_H_27_O_13_	Swertiamacroside	
17	8.3	463	C_21_H_19_O_12_	Hyperin	
18	8.7	727	C_38_H_31_O_15_	Epiafzelchin-epicatechin-*O*-methylgallate	455, 289, 271
19	9.4	455	C_23_H_19_O_10_	(−)-Epicatechin-3-(3”-*O*-methyl) gallate	289
20	9.5	561	C_30_H_25_O_11_	(Epi)afzelchin-(epi)catechin isomer B	543, 425, 289, 271
21	9.9	757	C_39_H_33_O_16_	Procyanidin B2-dimethylgallate	
22	10.7	741	C_39_H_33_O_15_	Epiafzelchin–epicatechin-*O*-dimethylgallate	
23	11.5	469	C_24_H_21_O_10_	Epicatechin-*O*-3,4-dimethylgallate	
24	12.3	463	C_21_H_19_O_12_	Isoquercitrin	
25	12.6	301	C_15_H_10_O_7_	Quercetin	

**Table 3:** Identification of bound phenolic compounds in buckwheat flours.

Peak	Retention Time	[M-H]	Molecular Formula	Compound
1	2.1	315	C_13_H_15_O_9_	2-hydroxy-3-*O*-β-d-glucopyranosylbenzoic acid
2	2.6	315	C_13_H_15_O_9_	Protocatechuic-4-*O*-glucoside acid
3	3.2	341	C_15_H_17_O_9_	Caffeic acid hexose isomer a
4	4.1	341	C_15_H_17_O_9_	Caffeic acid hexose isomer b
5	4.2	289	C_15_H_13_O_6_	Catechin
6	4.4	487	C_21_H_27_O_13_	Swertiamacroside isomer a
7	5.0	179	C_9_H_7_O_4_	Caffeic acid
8	5.5	289	C_15_H_13_O_6_	Epicatechin
9	6.3	197	C_9_H_9_O_5_	Syringic acid
10	6.8	447	C_21_H_19_O_11_	Orientin
11	6.9	163	C_9_H_7_O_3_	*p*-coumaric acid derivative
12	7.0	575	C_30_H_23_O_12_	Procyanidin A
13	7.5	317	C_15_H_9_O_8_	Myricetin
14	7.8	431	C_21_H_19_O_10_	Vitexin
15	7.9	609	C_27_H_29_O_16_	Rutin
16	7.9	441	C_22_H_17_O_10_	Epicatechin gallate
17	8.2	451	C_21_H_23_O_11_	Catechin-glucoside
18	8.2	487	C_21_H_27_O_13_	Swertiamacroside isomer b
19	8.7	727	C_38_H_31_O_15_	Epiafzelchin–epicatechin-*O*-methylgallate
20	9.3	163	C_9_H_7_O_3_	*p*-coumaric acid
21	9.4	455	C_23_H_19_O_10_	(−)-epicatechin-3-(3’’-*O*-methyl) gallate
22	11.5	469	C_24_H_21_O_10_	Epicatechin-*O*-3,4-dimethylgallate
23	12.3	463	C_21_H_19_O_12_	Isoquercitrin
24	12.6	301	C_15_H_10_O_7_	Quercetin

**Table 4:** Free phenolic compounds quantified in buckwheat meals/flours (mg/kg d.w.) determined by HPLC-MS.

Free Phenolic Compounds	Bran Meal	Middling Flour	Light Flour	De-hulled Grain Meal
2-hydroxy-3-*O*-β-Dglucopyranosylbenzoic acid	42.17 ^b^	78.22 ^a^	2.67 ^d^	32.71 ^c^
Protocatechuic-4-*O*-glucoside acid	79.69 ^b^	120.59 ^a^	2.93 ^d^	65.56 ^c^
Catechin-glucoside	23.87 ^b^	34.97 ^a^	1.88 ^d^	13.53 ^c^
Caffeic acid hexose	41.02 ^a^	37.39 ^b^	1.06 ^d^	30.95 ^c^
Catechin	20.40 ^a^	17.25 ^b^	1.36 ^d^	7.33 ^c^
Swertiamacroside	33.14 ^a^	22.81 ^b^	0.85 ^d^	9.84 ^c^
Caffeic acid	36.82 ^a^	22.35 ^b^	0.15^d^	0.96 ^c^
Epicatechin	69.56 ^a^	26.48 ^b^	2.60 ^d^	14.01 ^c^
(Epi)afzelchin-(epi) catechin isomer A	58.11 ^a^	35.49 ^b^	1.71 ^d^	20.06 ^c^
Orientin	5.18 ^a^	3.79 ^b^	0.02 ^d^	1.58 ^c^
Isorientin	4.61 ^a^	2.84 ^b^	<LOQ	0.82 ^c^
Vitexin	9.14 ^a^	6.26 ^b^	0.06 ^d^	2.02 ^c^
Rutin	214.99 ^a^	148.63 ^b^	7.03 ^d^	87.33 ^c^
Epicatechin-gallate	18.56 ^a^	7.82 ^b^	0.28 ^d^	5.22 ^c^
Epiafzelchin–epiafzelchin–epicatechin	20.37 ^a^	12.69 ^b^	0.84 ^d^	8.01 ^c^
Swertiamacroside	27.41 ^a^	20.92 ^b^	4.23 ^d^	9.47 ^c^
Hyperin	2.84 ^a^	1.59 ^b^	<LOQ	0.13 ^c^
Epiafzelchin–epicatechin-*O*-methyl gallate	76.84 ^a^	39.84 ^b^	1.00 ^d^	28.73 ^c^
(−)-Epicatechin-3-(3”-*O*-methyl) gallate	31.61 ^a^	17.77 ^b^	0.51 ^d^	15.18 ^c^
(Epi)afzelchin-(epi) catechin isomer B	25.04 ^a^	15.03 ^b^	0.47 ^d^	9.95 ^c^
Procyanidin B2-dimethylgallate	51.46 ^a^	29.22 ^b^	0.67 ^d^	21.06 ^c^
Epiafzelchin–epicatechin-*O*-dimethylgallate	216.94 ^a^	176.67 ^b^	13.11 ^d^	93.83 ^c^
Epicatechin-*O*-3,4-dimethylgallate	98.07 ^a^	8.05 ^c^	2.31 ^d^	39.10 ^b^
Isoquercitrin	1.41 ^a,b^	2.05 ^a^	0.54 ^d^	1.09 ^c^
Quercetin	33.21 ^a^	12.39 ^b^	0.06 ^d^	2.27 ^c^
Flavonoids	982.23 ^a^	598.23 ^b^	34.47 ^d^	371.25 ^c^
Phenolic acids	260.26 ^b^	302.28 ^a^	11.89 ^d^	149.49 ^c^
Sum	1242.49 ^a^	901.10 ^b^	46.36 ^d^	520.74 ^c^

Different letters in the same line show significant differences (*p* < 0.05), LOQ: Limit of quantification.

**Table 5:** Bound phenolic compounds quantified in buckwheat meals/flours (mg/kg d.w.) determined by HPLC–MS.

Bound Phenolic Compounds	Bran Meal	Middling Flour	Light Flour	De-Hulled Grain Meal
2-hydroxy-3-*O*-β-d-glucopyranosylbenzoic acid	23.02 ^b^	34.56 ^a^	6.19 ^c,d^	7.88 ^d^
Protocatechuic-4-*O*-glucoside acid	18.44 ^b^	25.50 ^a^	5.51 ^c^	5.95 ^c^
Caffeic acid hexose isomer a	5.52 ^b^	11.34 ^a^	0.67 ^c^	0.43 ^c,d^
Caffeic acid hexose isomer b	40.42 ^b^	56.73 ^a^	13.28 ^d^	26.35 ^c^
Catechin	207.74 ^a^	200.17 ^a^	54.67 ^c^	95.45 ^b^
Swertiamacroside	23.25 ^c,d^	31.84 ^a,b^	25.40 ^d^	33.66 ^a^
Caffeic acid	<LOQ	<LOQ	<LOQ	<LOQ
Epicatechin	59.08 ^b^	97.50 ^a^	34.67 ^d^	41.55 ^c^
Syringic acid	85.86 ^a^	43.57 ^b^	7.74 ^d^	35.62 ^c^
Orientin	0.46 ^a^	0.56 ^a^	0.19 ^c^	0.22 ^b^
*p*-coumaric acid derivative	9.65 ^a^	3.53 ^b^	1.39 ^d^	3.24 ^c^
Procyanidin A	8.82 ^a^	9.03 ^a^	0.95 ^c^	4.95 ^b^
Myricetin	4.12 ^a^	3.80 ^a^	2.06 ^b,c^	2.92 ^b^
Vitexin	4.22 ^a^	3.86 ^a^	0.67 ^c^	2.30 ^b^
Rutin	51.64 ^a^	45.19 ^b^	6.82 ^d^	33.71 ^c^
Epicatechin gallate	16.24 ^a^	15.57 ^a^	4.21 ^c^	10.75 ^b^
Catechin-glucoside	16.48 ^a^	17.51 ^a^	1.04 ^c^	13.26 ^b^
Swertiamacroside	39.40 ^a^	32.37 ^b^	23.52 ^d^	30.43 ^c^
Epiafzelchin–epicatechin-*O*-methylgallate	28.04 ^a^	27.81 ^a^	3.57 ^c^	9.72 ^b^
*p*-coumaric acid	3.96 b	6.91 ^a^	0.67 ^d^	2.74 ^c^
(−)-epicatechin-3-(3”-*O*-methyl) gallate	6.09 ^a^	6.05 ^a^	2.06 ^c^	4.17 ^b^
Epicatechin-*O*-3,4-dimethylgallate	4.65 ^a^	4.11 ^a^	0.50 ^c^	1.78 ^b^
Isoquercitrin	6.06 ^a^	5.89 ^a^	1.03 ^c^	3.64 ^b^
Quercitrin	26.64 ^a^	21.05 ^b^	10.94 ^d^	18.78 ^c^
Flavonoids	440.29 ^b^	458.11 ^a^	123.37 ^d^	243.20 ^c^
Phenolic acids	249.52 ^a^	246.35 ^b^	84.37 ^d^	146.31 ^c^
Total	689.81 ^b^	704.47 ^a^	207.74 ^d^	389.51 ^c^

Different letters in the same line show significant differences (*p* < 0.05), LOQ: Limit of quantification.

**Table 6:** Total content of flavonoids, phenolic acids, and phenolic compounds in buckwheat flours. Results are expressed as mg/kg d.w.

	Flavonoids	Phenolic Acids	Total
Bran meal	1422.52 ^a^	509.78 ^b^	1932.30 ^a^
Middling flour	1056.94 ^b^	548.63 ^a^	1605.57 ^b^
Light flour	157.84 ^d^	96.261 ^d^	254.10 ^d^
De-hulled grain meal	614.46 ^c^	295.80 ^c^	910.25 ^c^

Different letters in the same column show significant differences (*p* < 0.05).
